# Indirect Calorimetry as an Instrument of Research to Identify the Effect of Hypermetabolism in Critical Patients’ Prognosis

**DOI:** 10.7759/cureus.17784

**Published:** 2021-09-07

**Authors:** Grimanesa Sousa, Inês Mendes, Luís Tavares, Rita Brotas Carvalho, Manuela Henriques, Humberto Costa

**Affiliations:** 1 Department of Intensive Care Medicine, Hospital do Divino Espírito Santo de Ponta Delgada, Ponta Delgada, PRT; 2 Department of Endocrinology and Nutrition, Hospital do Divino Espírito Santo de Ponta Delgada, Ponta Delgada, PRT

**Keywords:** hypermetabolism, indirect calorimetry, critical illness, energy expenditure, nutrition

## Abstract

Background: Energy expenditure (EE) evaluation in Intensive Care Unit (ICU) patients can be very challenging. Critical illness is characterized by great variability in EE, which is influenced by the disease itself and the effects of treatment. Indirect calorimetry (IC) is currently the gold standard to measure EE in Intensive Care Unit (ICU) patients. However, calorimeters are not widely available, and predictive formulas (PF) are still commonly used, leading to under or overfeeding and deleterious consequences.Important metabolic changes occur and catabolism becomes prominent in critically ill patients.Both hyper and hypometabolism can be observed, but hypermetabolic patients appear to have higher mortality rates compared to metabolically normal patients. This study aimed to assess hypermetabolism incidence and compare clinical outcomes between hypermetabolic and normometabolic patients in ICU.

Methods: A single-center, retrospective, and observational study was conducted in the ICU of the Hospital do Divino Espírito Santo in Ponta Delgada, between August 2018 and February 2021. Only invasively mechanically ventilated patients were included. Resting energy expenditure (REE) was predicted by 25 kcal/kg/day formula to obtain predicted resting energy expenditure (PREE), and REE was measured by IC to obtain measured resting energy expenditure (MREE). According to their metabolic state (PREE/MREE), patients were divided into hypermetabolic (≥1.3) and normometabolic (<1.3). To determine the limits of agreement between PREE and MREE, we performed a Bland-Altman (BA) analysis. Baseline characteristics, severity criteria, nutritional status, and main diagnosis on admission were compared. The primary outcome considered was 30-day mortality. Other outcomes such as the ICU length of stay (LOS), in-hospital LOS, and length of invasive ventilation were also evaluated.

Results: Among the 80 ICU patients included in the final analysis, 67 patients were normometabolic (83.4%). Patients admitted due to pneumonia were more hypermetabolic, 8 (61.5%) vs. 10 (14.9%); p<0.001. Hypermetabolism was found also in patients admitted due to sepsis/septic shock, 7 (53.8%) vs. 16 (23.9%); p=0.029. Hypermetabolic patients had lower body mass index (22.5 [interquartile range (IQR): 21.5-24.9] vs. 27.7 [IQR: 25.0-32.4] kg/m^2^; p=0.001) and higher MREE (2715.0 [2399.0-3090.0] vs. 1690.0 [1410.0-2190.0] kcal/day; p<0.001). Bland-Altman analysis showed a mean difference of -5.6 ± 744.7 Kcal/day between the PREE and MREE by IC. No statistically significant difference was found between the two groups, neither in 30-day mortality nor in the other outcomes considered.

Conclusions: Hypermetabolism was not seen to present a greater risk of death in mechanically ventilated patients in ICU. Lower BMI, sepsis/septic shock, and pneumonia appear to be associated with a hypermetabolic state.

## Introduction

The total energy expenditure (TEE) is defined as the total amount of energy humans need to function. It is divided into three components: basal energy expenditure (BEE), diet-induced thermogenesis (DIT), and activity-related energy expenditure (AEE). BEE and DIT combined represent the resting energy expenditure (REE), which is defined as all energy requirements in the body’s basal metabolism to maintain vital functions while inactive [1,2]. In critically ill patients, REE will closely reflect TEE because of minimal physical activity [3].

In critical illness, important changes occur in energy utilization and substrate metabolism [4], leading to a prominent stimulation of catabolic pathways and energy expenditure [5]. Theoretically, this can exert a profound influence on energy consumption and also increase REE observed in Intensive Care Unit (ICU) patients [6]. Critical illness is characterized by great variability in the REE, which is influenced not just by the disease itself but also by the effects of treatment, anthropometrics, nutritional status, (in)activity, and environment [3,7,8]. Hypermetabolism is frequently detected in ICU, mediated by some conditions like sepsis, burns, and hyperthermia. Nevertheless, both hyper or hypometabolism can be observed [9].

The predictive energy requirements equations like the Harris and Benedict prediction [10] and others [11] have been used conventionally for decades to assess the caloric requirements of critically ill patients. However, inaccuracies ranging up to 60% in these equations led to the need for more accurate measures such as indirect calorimetry (IC) for caloric requirement assessment [12]. Actually, IC is considered the gold standard to measure REE and caloric needs in critically ill patients at the bedside [13]. Caloric needs estimation by IC is based on oxygen (O_2_) consumption and carbon dioxide (CO_2_) generation in critically ill patients [13]. Its use has been strongly recommended by the recent European Society for Clinical Nutrition and Metabolism (ESPEN) and American Society for Parenteral and Enteral Nutrition (ASPEN) guidelines [3,7,14,15]. However, calorimeters are not widely available, and predictive formulas are still commonly used, leading to under or overfeeding and deleterious consequences [16].

Hypermetabolism is a common complication of critical illness mediated by the immune system, which can be affected by damaged tissue rupture and/or pathogenic microorganisms and the entry of their toxins into the bloodstream, as well as the body’s response (for example, hormone and cytokine release). Due to this situation, hypermetabolic patients often seem to have higher mortality rates than metabolically normal patients [9,17].

The present study was designed to evaluate the hypothesis that hypermetabolism leads to a mortality increase in ICU patients.

## Materials and methods

This single-center, retrospective, and observational study was carried out between August 2018 and February 2021 in the ICU of Hospital do Divino Espírito Santo in Ponta Delgada, Portugal. Only mechanically ventilated patients were included. Patients ventilated with a fraction of inspiration oxygen (FiO2) above 60% and positive end-expiratory pressure (PEEP) above 12 cmH2O were excluded, because these parameters can profoundly affect the accuracy of IC testing [18]. Hemodynamic and respiratory stability were also needed. Predicted REE (PREE) was estimated using the weight-based predictive equation (25 kcal/kg/day) proposed by ESPEN and ASPEN guidelines [14,15]. The measured REE (MREE) of each patient was determined using a portable calorimeter, CARESCAPE ® Monitor B650 (General Electric Co., Boston, USA), for 24 hours.

Data collection

Baseline characteristics, severity scores [Acute Physiology And Chronic Health Evaluation II (APACHE II), Simplified Acute Physiology Score II (SAPS II), and Sequential Organ Failure Assessment (SOFA)], potentially relevant comorbidities, and main diagnosis on admission were recorded. Nutritional data like body mass index (BMI, kg/m^2^), Nutrition Risk in Critically ill (NUTRIC) score [without interleukin (IL)-6], and plasmatic albumin at admission were also registered. The primary outcome considered was 30-day mortality. Other outcomes such as the ICU length of stay (LOS), in-hospital LOS, length of invasive mechanical ventilation (IMV), and nosocomial infections were also evaluated.

Statistical analysis

Based on the ratio MREE/PREE, patients were divided into two groups [17]: hypermetabolic group if MREE/PREE ≥ 1.3 or normometabolic group if MREE/PREE <1.3. A Bland-Altman analysis was performed to determine the limits of agreement between MREE and PREE. The software KNIME Analytics Platform® version 4.1.2 (KNIME Inc., Zurich, Switzerland) was used for data analysis. Categorical data are expressed in *n* (%) and compared by the use of the chi-squared test. According to the one-sample Kolmogorov-Smirnov test, the continuous variables don't follow a normal distribution. Therefore, all of them were expressed by their median (interquartile range [IQR]). The numerical continuous variables were compared using the Wilcoxon test. All statistical tests were two-tailed, and significance was defined as *p*-value <0.05.

## Results

Eighty critically ill patients were included. Overall, 67 (83.8%) patients were in the normometabolic group and 13 (16.3%) patients were in the hypermetabolic group. The baseline characteristics of the study sample are described in Table 1.

**Table 1:** Baseline characteristics of the studied population. APACHE II: Acute Physiology and Chronic Health Evaluation II; COPD: Chronic Obstructive Pulmonary Disease; SAPS II: Simplified Acute Physiology Score II; SOFA: Sequential Organ Failure Assessment; IQR: interquartile range.

Study characteristics	Overall (n=80)	Hypermetabolic (n=13)	Normometabolic (n=67)	p value
Age, median [IQR], years	59.5 [44.5-68.0]	52.0 [43.0-59.0]	61.0 [45.5-68.5]	0.127
Male, n (%)	58 (72.5)	11 (84.6)	47 (70.1)	0.285
Comorbidities				
Diabetes, n (%)	18 (22.2)	1 (7.7)	17 (25.4)	0.162
COPD, n (%)	12 (15.0)	1 (7.7)	11 (16.4)	0.420
Hearth failure, n (%)	8 (10.0)	0 (0.0)	8 (11.9)	0.515
Chronic kidney disease, n (%)	3 (3.8)	1 (7.7)	2 (3.0)	0.414
Severity				
APACHE II score, median [IQR]	17.5 [13.0-22.0]	15.0 [13.0-20.0]	18.0 [13.5-22.0]	0.510
SAPS II score, median [IQR]	44.0 [36.0-52.5]	42.0 [37.0-58.0]	45.0 [34.8-52.0]	0.937
SOFA score median [IQR]	8.0 [5.0-9.0]	8.0 [7.0-9.0]	8.0 [4.5-9.0]	0.454
Type of admission				
Medical, n (%)	45 (56.2)	9 (69.2)	36 (53.7)	0.303
Surgical, n (%)	35 (43.8)	4 (30.8)	31 (46.3)	
Main diagnosis				
Sepsis and septic shock, n (%)	23 (28.8)	7 (53.8)	16 (23.9)	0.029
Neurocritical, n (%)	24 (30.0)	3 (23.1)	21 (31.3)	0.552
Pneumonia, n (%)	18 (22.5)	8 (61.5)	10 (14.9)	<0.001
Haemorrhagic shock, n (%)	11 (13.8)	1 (7.7)	10 (14.9)	0.488
Cardiac arrest, n (%)	8 (10.0)	0 (0.0)	8 (11.9)	0.515

Male gender was prevalent (n=58; 72.5%), but we did not find any statistically significant difference between groups (p=0.285). The global median age was 59.5 [IQR: 44.5-68.0] years. Hypermetabolic patients were older than normometabolic patients, although without statistical significance (p=0.127). No statistically significant difference was found between the two groups in all the comorbidities studied (diabetes, chronic obstructive pulmonary disease [COPD], heart failure, or chronic kidney disease). Also, no statistically significant difference was found between the two groups in terms of severity scores: APACHE II (p=0.510), SAPS II (p=0.937), and SOFA (p=0.454).

Most of the patients were admitted due to medical causes (56.2%), but no statistically significant difference was found concerning the two groups in terms of metabolism (p=0.303). Patients admitted due to pneumonia (n=18; 22.5%) were more hypermetabolic (61.9% vs 14.9%) with statistical significance (p<0.001). We also found a statistically significant difference between the two groups in patients admitted due to sepsis/septic shock, where septic patients were more hypermetabolic (53.8% vs. 23.9%; p=0.029). No statistically significant difference was found between the two groups in admissions due to other conditions.

The nutritional data of the sample is showed in Table 2. The median NUTRIC score (without IL-6) was 4.0 [IQR: 2.0-7.0]. Hypermetabolic group had a lower BMI compared to normometabolic group, 22.5 [IQR: 21.5-24.9] vs. 27.7 [IQR: 25.0-32.4] kg/m^2^; p=0.001. Hypermetabolic patients had also a lower PREE than normometabolic: 1500.0 [IQR: 1300.0-1875.0] vs. 1925.0 [IQR: 1712.0-2375.0] kcal/day; p=0.002. In median, MREE was determined (by IC) on day 4 of admission [IQR: 2.0-7.0]. MREE was higher in hypermetabolic patients, 2715.0 [2399.0-3090.0] vs. 1690.0 [1410.0-2190.0] kcal/day, with statistical significance (p<0.001).

**Table 2:** Nutritional data. BMI: Body mass index; NUTRIC score: Nutrition Risk in Critically ill; RASS: Richmond Agitation-Sedation Scale; IQR: interquartile range; PREE: Predicted resting energy expenditure; MREE: Measured resting energy expenditure Albumin reference range: 3.5-5.5 g/dL

	Overall (n=80)	Hypermetabolic (n=13)	Normometabolic (n=67)	p-value
Nutritional state				
BMI, Kg/m^2^	27.3 [24.1-30.8]	22.5 [21.5-24.9]	27.7 [25.0-32.4]	0.001
NUTRIC score	4.0 [3.0-6.0]	4.0 [2.0-6.0]	4.0 [3.0-6.0]	0.822
Albumin level, median [IQR], g/dL	2.5 [2.0-2.8]	2.4 [2.3-2.8]	2.5 [2.0-2.9]	0.676
Rest energy expenditure				
PREE, median [IQR] (kcal/day)	1875.0 [1625.0-2250.0]	1500.0 [1300.0-1875.0]	1925.0 [1712.0-2375.0]	0.002
MREE, median [IQR] (kcal/day)	1781.0 [1474.0-2420.0]	2715.0 [2399.0-3090.0]	1690.0 [1410.0-2190.0]	<0.001
Indirect calorimetry, day	4.0 [2.0-7.0]	4.0 [3.0-4.0]	4.0 [2.0-7.5]	0.854
Nutritional support				
Enteral, n (%)	60 (75.0)	11 (84.6)	49 (73.1)	0.382
Parenteral, n (%)	17 (21.2)	2 (15.4)	15 (22.4)	0.572
Sedation				
Profound sedation (RASS-5), n (%)	51 (63.8)	9 (69.2)	42 (62.7)	0.653
Neuromuscular blockage, n (%)	8 (10.0)	2 (15.4)	6 (9.0)	0.480

**Figure 1:**
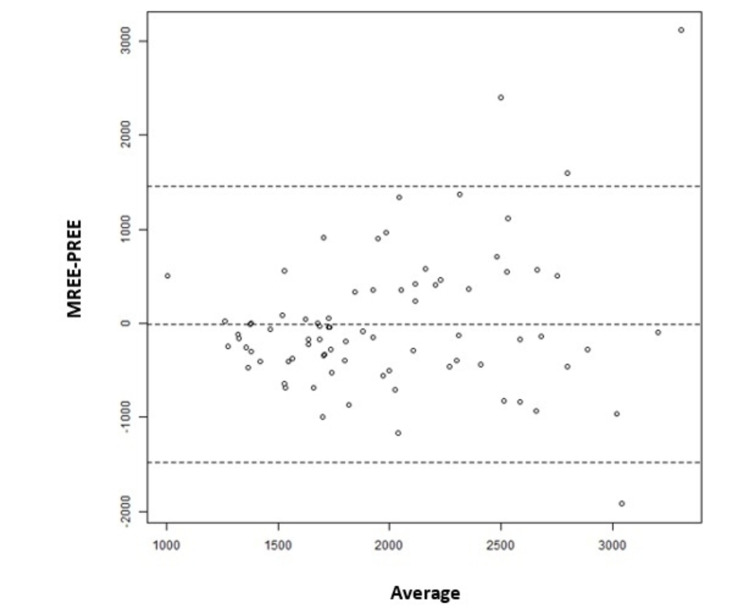
Bland-Altman analysis between MREE by IC and PREE based on weight formula (25 kcal/Kg/day). The x-axis shows the average resting energy expenditure  (REE) by the two methods, measured resting energy expenditure (MREE) and predicted resting energy expenditure (PREE) (kcal/day). The y-axis shows the difference in REE between the two methods, MREE and PREE (kcal/day). In the case of both the methods of measurement having a good agreement, the graph points are centered on the “0” y-axis, regardless of the average REE.

Bland-Altman analysis showed a mean difference of -5.6 ± 744.7 kcal/day between the PREE considering the 25 kcal/kg/day formula and the MREE by IC (Figure 1).

Overall, 15 patients died in 30 days after admission (18.8% observed mortality). No statistically significant difference in the 30-day mortality was found between groups as the first outcome studied (p=0.662). In our study, the other outcomes like in-hospital mortality, in ICU or in-hospital LOS, length of IMV, or nosocomial infections, failed to show any difference between the two groups. The study outcomes are shown in Table 3.

**Table 3:** Outcomes of the study. ICU: Intensive care unit; LOS: Length of stay; IQR: interquartile range; IMV: Invasive mechanical ventilation

Study outcomes	Overall (n=80)	Hypermetabolic (n=13)	Normometabolic (n=67)	p-value
30-day mortality, n (%)	15 (18.8)	3 (23.1)	12 (17.9)	0.662
In-hospital mortality, n (%)	11 (13.8)	3 (23.1)	8 (11.9)	0.286
ICU LOS, median [IQR], days	16.0 [9.8-25.2]	15.0 [8.0-20.0]	16.0 [10.5-26.0]	0.371
Hospital LOS, median [IQR], days	37.0 [22.5-52.8]	37.0 [15.0-43.0]	37.0 [23.5-58.0]	0.531
IMV, median [IQR], days	11.0 [6.9-16.6]	7.5 [6.3-14.3]	11.0 [7.0-17.0]	0.332
Nosocomial infections, n (%)	39 (48.8)	4 (30.8)	35 (52.2)	0.156

## Discussion

The study was conducted on the hypothesis that hypermetabolism may be associated with poor prognosis in ICU patients. However, our results failed to show an association between hypermetabolism and all the analyzed outcomes.

In critical patients, important changes occur in energy expenditure and substrate metabolism. Similarly, critical illnesses seem to influence energy use and increase REE significantly [6]. Studies analyzing different pathologies observed a high prevalence of hypermetabolism in ICU patients [17,19]. Nevertheless, the prevalence of normometabolic patients observed in our study (82.6%) contrasts with the statement that critically ill patients are hypermetabolic in most cases. However, the definition of hypermetabolism is not consensual, which makes it difficult to compare available data [9,17,19,20]. Less strict parameters were used in other studies, diagnosing hypermetabolism when PREE/MREE >1.15. Probably, for that reason, they found a higher prevalence of hypermetabolic patients (83%) [19]. One study assessing septic patients in ICU found a lower prevalence of hypermetabolism (54.8%) defined when studied patients had PREE/MREE ≥ 1.3 [17]. That was the cut-off used in our study to define hypermetabolism, and only 13 patients (16.3%) met these criteria.

The reduction of metabolic and systemic stress, with a consequent decrease of energy requirements, can be explained by the effect of widely used drug therapies in ICU such as sedatives, analgesics, and muscle blockers [21-23]. In fact, in the presented study, 63.8% of overall patients were deeply sedated (RASS-5) during IC measurement. The fact that IC measurement was performed on day four of ICU admission (according to the median) can have also contributed to the low rate of hypermetabolic patients. It could lead to less hypermetabolism because patients seem to be under a hypermetabolic state during the early phase of critical illness [17].

The hypermetabolic group had a higher proportion of males (84.7 vs. 70.1%). Although without statistical significance (p=0.285), our findings are consistent with the published data that showed that female patients had a lower REE despite the same critically ill condition [24]. Our study showed that hypermetabolic patients were younger than normometabolic, although without statistical significance (p=0.127). According to the literature, a progressive decline in REE of about 1-2% per decade of life is observed, and this decline is mostly explained by changes in body composition [25,26].

Respiratory chronic conditions, as described in elderly patients with COPD, were associated with the increased REE that seems to be independent of the severity of the pulmonary obstruction [27]. However, we did not find any statistically significant difference between groups, neither in COPD patients nor in other comorbidities studied.

Some conditions like acute kidney injury (AKI) and sepsis were associated with hypermetabolism [17,28]. This is consistent with our findings since septic patients were found to be more hypermetabolic (p=0.029). In the presented study, we also identified a relevant association between pneumonia and hypermetabolic state. These findings are consistent with a recent study in coronavirus diseases 2019 (COVID-19) patients in whom viral pneumonia lead to a persistent hypermetabolic state [29].

We found that lower BMI was associated with the presence of hypermetabolism (p=0.001). This association may be observed because the 25 kcal/kg/day formula (using real weight) may not be the best tool to estimate PREE. This fact is more evident in overweight patients as it overestimates REE, thus categorizing these patients as normometabolic [30]. Nevertheless, using Bland-Altman analysis, we show a good correspondence between the 25 kcal/kg/day formula and the MREE by IC; however, with a standard deviation of 744.7 kcal/day. This means that for some patients PREE by the 25 kcal/kg/day formula can over or underestimate their real REE. It shows that for underweight or obese patients the PREE weight-based formula may not be suitable, but it could be for healthy weight patients. These results support the guideline’s indications to use IC as the gold standard to measure REE [14,15].

Although small and heterogenic, the analyzed sample is one of the largest published literature using IC in critically ill patients. However, some limitations of this study must be elucidated, like their retrospective design, and their unicentric nature. The variability on the day of the IC measurement meant the patients were at different stages in the course of their disease.

## Conclusions

Our study failed to show a difference in 30-day mortality in either normometabolic or hypermetabolic patients. However, it can be concluded that patients admitted due to pneumonia and sepsis/septic shock were more hypermetabolic. The hypermetabolic patients had a lower BMI and a higher MREE by IC. A good correspondence between MREE by IC and PREE using the 25 kcal/kg/day formula in critically ill patients was also found.
